# Workplace Bullying and Mental Health: A Meta-Analysis on Cross-Sectional and Longitudinal Data

**DOI:** 10.1371/journal.pone.0135225

**Published:** 2015-08-25

**Authors:** Bart Verkuil, Serpil Atasayi, Marc L. Molendijk

**Affiliations:** 1 Institute of Psychology, Leiden University, Leiden, The Netherlands; 2 Leiden Institute for Brain and Cognition, Leiden University Medical Center, Leiden, The Netherlands; 3 Skils, Leiden, The Netherlands; University of Geneva, SWITZERLAND

## Abstract

**Background:**

A growing body of research has confirmed that workplace bullying is a source of distress and poor mental health. Here we summarize the cross-sectional and longitudinal literature on these associations.

**Methods:**

Systematic review and meta-analyses on the relation between workplace bullying and mental health.

**Results:**

The cross-sectional data (65 effect sizes, *N* = 115.783) showed positive associations between workplace bullying and symptoms of depression (*r* = .28, 95% *CI* = .23–.34), anxiety (*r* = .34, 95% *CI* = .29–.40) and stress-related psychological complaints (*r* = .37, 95% *CI* = .30–.44). Pooling the literature that investigated longitudinal relationships (26 effect sizes, *N* = 54.450) showed that workplace bullying was related to mental health complaints over time (*r* = 0.21, 95% *CI* = 0.13–0.21). Interestingly, baseline mental health problems were associated with subsequent exposure to workplace bullying (*r* = 0.18, 95% *CI* = 0.10–0.27; 11 effect sizes, *N* = 27.028).

**Limitations:**

All data were self-reported, raising the possibility of reporting- and response set bias.

**Conclusions:**

Workplace bullying is consistently, and in a bi-directional manner, associated with reduced mental health. This may call for intervention strategies against bullying at work.

## Introduction

Affective disorders, such as major depression and anxiety disorders, are highly prevalent mental disorders that place a great burden on individuals as well as on society [1]. Estimations are that each year, 7.8% of the European population suffers from a mood disorder and 14% from an anxiety disorder [2]. An even larger part of the population is currently severely worried and emotionally exhausted and suffers from stress-related psychological complaints that do not fully justify a formal diagnosis [3]. Yet, these people are at high risk of developing an anxiety or depressive disorder [3]. Given the extensive mental, physical and economic burden associated with these mental health problems it is pivotal to identify factors that are associated with increased risk of these problems.

When asked, 33% of the patients with mood disorders attribute their mental problems to their work situation [4], making problems at work the most common self-reported cause of depression. That work has an impact on mental health is not surprising, since people spend most of their daily lives at work. Work provides meaning, income, and social relationships, but it can also cause stress [5]. The most extensive studied forms of work-related stress factors are perceived job control and demands [6] and effort-reward imbalances [7]. Yet, other work related factors are believed to influence mental health as well. Amongst these is workplace bullying. Studies suggest that between 2 and 30% of the working population has experienced bullying at work [8].

The concept of workplace bullying entails situations in the workplace where an employee persistently and over a long time perceives him- or herself to be mistreated and abused by other organization members, and where the person in question finds it difficult to defend him/herself against these actions (definition provided by: [9]). Workplace bullying may be related specifically to one’s tasks and can take the form of unreasonable deadlines, meaningless tasks, or excessive monitoring of work [10]. Workplace bullying may also be person-related and take the form of gossiping, verbal hostility, persistent criticism, or social exclusion [10–12]. A critical aspect of workplace bullying, shared by the manifold operationalizations that exist, is that is not limited to one single event, but that it is a persistent experience throughout one’s working days [10–12].

Consistent with stress theories, workplace bullying has been recognized as a main source of distress that is associated with subsequent health and decreased well-being [13], to lowered job satisfaction and performance [9,14], reduced commitment [9], and higher levels of sickness absenteeism [15,16]. In addition, workplace bullying has been associated with psychotropic drug use [17].

A driving force between workplace bullying and the above-mentioned variables may be that workplace bullying causes mental health problems [18]. To our knowledge, there are four meta-analyses synthesizing the evidence of the relation between workplace bullying and mental health outcomes. The first of these, somewhat preliminary due to a small number of studies, comes from Hershcovis [19]. Hershcovis compared the consequences of workplace bullying, abusive supervision, interpersonal conflict, incivility and social undermining on psychological and physical well-being, turnover intent, and job satisfaction. A second meta-analysis summarized the cross-sectional and longitudinal data on the relation between workplace bullying and mental- and physical health, as well as job-related outcomes [9]. Recently, two new meta-analyses were published. In one the longitudinal relation between workplace bullying and mental health is summarized [20], but this paper is only available in the Norwegian language. In the other, the evidence for a link between school- and workplace bullying and symptoms of post-traumatic stress is summarized [21]. All these studies provided support for a relation between workplace bullying and mental health.

Yet, since the publication of the meta-analysis in 2012, many new studies have appeared, especially longitudinal ones. This warrants an updated meta-analysis in order to provide researchers, clinicians, and policy makers with a complete overview on the relation between bullying at work and mental health. Furthermore, from earlier studies it appears that there is heterogeneity in between-study effect-size estimates (e.g., [9]). We wish to elucidate whether population- and methodological characteristics of individual studies (the age of the bullied person, gender distribution of the sample, the measurement method of bullying, type of work of the bullied person, year of publication and methodological quality rating of the study) may explain this heterogeneity. We choose these moderators because (1) several of these variables have been related to mental health (eg., [22,23]) and (2) they were available in most studies.

In the present study, we provide an integrated picture of the relation between workplace bullying and mental health problems, including both cross-sectional as well as prospective studies. We examined the relation between workplace bullying and mental health, consisting of three categories, namely (1) symptoms of depression, (2) symptoms of anxiety, and (3) stress-related psychological complaints, such as negative affect and emotional exhaustion. Finally, we explore the possibility that baseline mental health problems are associated with subsequent exposure to workplace bullying.

## Method

### Search strategy

To identify eligible studies, we searched electronic databases (PubMed and PsychINFO up to and including February 2015 using the following keyword profile: (((work* OR job OR occupational OR workplace) AND (mobb* OR bulli* OR bully))) AND (health OR depress* OR anxiety OR stress OR mood OR psychological OR well-being OR traumatic OR PTSD OR sadness). In addition, the reference lists of the included papers were checked for eligible articles and a supplementary backward search was conducted.

### Inclusion criteria

**Fig 1:**
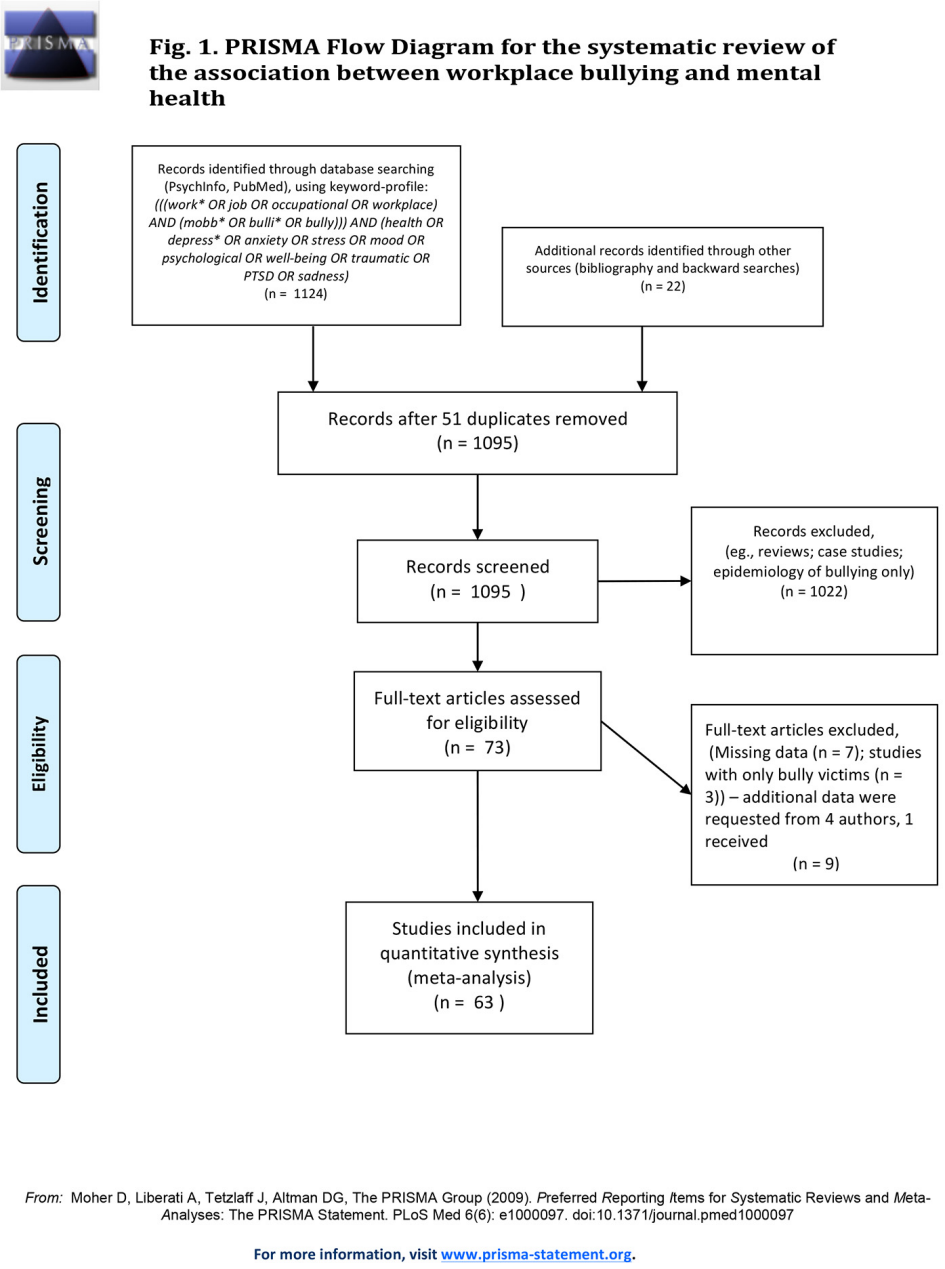
PRISMA flow diagram for the systematic review of the association between workplace bullying and mental health.

To be included, studies had to report on the association between workplace bullying and depression and/or anxiety, psychological distress or mental health problems in general. Workplace bullying had to be operationalized in line with the definition described in the introduction section of this paper. **S1 Table** lists the questionnaires that were used to gauge on the outcomes of interest. Inclusion was not dependent on year of publication. Similar to Nielsen and Einarsen´s [9] inclusion criteria, studies reporting on non-recurring or sporadic incidents of interpersonal harassment or violence at work were excluded. Furthermore, we focused on samples that were derived from the general working population, and excluded studies that specifically recruited self-labeled victims of bullying who were seeking treatment at specialized clinics [24,25]. Finally, studies had to report zero-order correlations between workplace bullying and the outcome variables described above, or provide the necessary data after request by e-mail. Studies for which missing information could not be obtained from the corresponding author or studies reporting inappropriate data (e.g. reviews, case studies, *etcetera*) were not included in the analyses. The search revealed approximately 1.100 potentially eligible published papers. We refer to **Fig 1** for a flow-chart and S1 Table for more information on the search process and the decisions to include or exclude articles.

### Data extraction

All effect sizes were converted to standardized Pearson product-moment correlation coefficients *r*, as most studies reported correlation coefficients. In case multiple correlation coefficients were reported in a study, for instance because of multiple measures of the same outcome, we averaged the outcomes to yield a single study-wide correlation coefficient. Furthermore, when reporting multiple outcomes of interest (e.g., depression and anxiety), the average correlation was calculated and used in the first overall analysis (relation between workplace bullying and mental health).

In addition to mental health outcomes of workplace bullying, data were extracted on (**I**) demographic characteristics: mean age, percentage females, ethnicity, occupation, and country in which the study was performed and (**II**) methodological characteristics such as measurement tools for predictor and outcome variables and their validity.

The methodological quality of each included study was assessed using the Newcastle-Ottawa Scale (N-OS [26]), with quality of an individual study defined as the frequency of criteria that it met. **S2 Table** provides information on quality assessment, including total scores for each individual study by the two independent raters (BV and MM). For the studies that we included in our analysis, the average quality score was 4.36 (SD = 0.93). The agreement between the independent raters was high (Cohen’s kappa = 0.76, standard error = 0.03).

### Statistical analyses

Analyses were carried out using the metafor package in R [27]. Statistical significance of the pooled *r* was assessed using a *Z*-test at *P* < .05. Heterogeneity between the studies was anticipated and thus the random effects model was used [28]. I^2^ was used to measure heterogeneity.

The potential moderating effect of mean age and gender distribution of the sample, symptom clusters, measurement methods, job type, year of publication was assessed by entering these variables as continuous or categorical predictors into the random effects model. The potential presence of publication bias was assessed by means of funnel plot inspection and random regression analyses were used to test for funnel plot asymmetry [29]. In case of a publication bias, Duval and Tweedie´s trim-and-fill procedures [30] were performed to assess the pooled effect size while taking into account publication bias.

## Results

### Description of samples

The 48 samples–derived from 42 articles [8,18,31–70]–that were included in the cross-sectional analyses are described in **Table 1**. From these 48 samples, 65 effect sizes were extracted and used in the meta-analyses. In sum, the number of the participants included in the individual studies ranged from *n* = 107 to *n* = 42898 (*M* = 3490, *SD* = 8751). In 31 (69%) out of 45 studies that reported the gender distribution of the sample, the majority of participants were female. The mean age of the entire samples ranged from 26 to 53 years (*M* = 40, *SD* = 6). With regard to the field of work, the larger parts of the participants were either derived from general working samples (47%) or from healthcare employees (32%). As a measurement method for workplace bullying, the NAQ [39] was used most frequently (i.e., in 42% of the studies).

**Table 1:** Basic demographic and methodological characteristics of the included studies.

Author, year	Analysis ^A^	*N* (*n* bullied/non bullied)	% Female	Mean age	Country
Quine, 1999	ANX, DEP	1,100 (421/679)	84	35	UK
Mikkelsen and Einarsen, 2001	ANX, DEP	571 (18/499)	82	40	Denmark
Mikkelsen and Einarsen, 2002	MHEALTH	433 (381/52)	45	39	Denmark
Vartia and Hyyti, 2002	STRESS	896 (161/735)	20	40	Finland
Quine, 2003	STRESS	594 (220/374)	50	NK	UK
Bilgel *et al*., 2006	ANX, DEP	944 (483/461)	50	35	Turkey
Hansen *et al*., 2006	ANX, DEP, STRESS	437 (22/415)	64	51	Sweden
Lee *et al*., 2006	STRESS	180 (NK/NK)	60	38	Canada
Niedhammer *et al*., 2006	DEP	7,694 (763/6,931)	59	40	France
Moreno-Jiménez *et al*., 2007	ANX	120 (NK/NK)	33	47	Spain
Mathisen *et al*., 2008	STRESS, BO	207 (14/193)	39	26	Norway
Sa and Fleming, 2008	DEP, STRESS, BO	107 (14/93)	85	36	Portugal
Einarsen *et al*., 2009	STRESS	5,288 (NK/NK)	48	40	UK
Bond *et al*, 2010	PTSD	139 (NK/NK)	25	31	Australia
Hauge *et al*, 2010	ANX, DEP	2,242 (NK/NK)	50	44	Norway
Laschinger *et al*., 2010	STRESS, BO	415 (137/278)	95	27	Canada
Balducci *et al*, 2011	PTSD	609 (NK/NK)	49	NK	Italy
Glasø *et al*, 2011	STRESS	462 (316/146)	14	45	Norway
Hansen *et al*, 2011	DEP, STRESS	1,922 (139/1,783)	67	49	Sweden
Kingdom and Smith, 2011	ANX, DEP	280 (NK/NK)	NK	NK	UK
Law *et al*., 2011	STRESS	220 (NK/NK)	53	41	Australia
Rodríguez-Muñoz *et al*., 2011	STRESS	4,068 (NK/NK)	44	40	Belgium
Vie *et al*., 2011	STRESS	904 (116/788)	14	49	Norway
Dehue *et al*., 2012	DEP, STRESS	361 (139/222)	45	43	The Netherlands
Glasø and Notelaers, 2012	STRESS	5,520 (NK/NK)	42	41	Belgium
Hogh *et al*., 2012	PTSD	1010 (NK/NK)	NK	48	Denmark
Laschinger and Grau, 2012	DEP, STRESS, BO	165 (NK/NK)	93	28	Canada
Rodwell and Demir, 2012	DEP, STRESS	441 (176/274)	99	45	Australia
Rodwell *et al*., 2012	STRESS	150 (34/116)	93	40	Australia
Carter *et al*., 2013	STRESS	2,950 (575/2,375)	72	41	UK
Demir *et al*., 2013	DEP, STRESS	166 (52/114)	86	NK	Australia
Gardner *et al*., 2013	STRESS	2,950 (575/2,375)	NK	NK	New Zealand
Laschinger and Nosko, 2013	PTSD	875 (NK/NK)	94	46	Canada
Trepanier *et al*., 2013	STRESS, BO	1,179 (NK/NK)	91	43	Canada
Bardakçi and Günüşen., 2014	STRESS	284 (62/222)	100	34	Turkey
Cassidy *et al*., 2014	STRESS	2,068 (NK/NK)	68	32	UK
Khubchandani and Price, 2014	STRESS	17,524 (NK/NK)	52	43	USA
Kostev *et al*., 2014	ANX, DEP	5,250 (NK/NK)	67	41	Germany
Malik and Farooqi, 2014	PTSD	300 (NK/NK)	100	40	Pakistan
Malinauskiene and Einarsen, 2014	PTSD	323 (110/213)	82	53	Lithuania
Tuckey and Neal, 2014	STRESS, BO	221 (NK/NK)	77	36	Australia
Niedhammer *et al*., 2015	ANX, DEP	42,898 (10,752/32,146)	43	NK	France
**Longitudinal studies**					
Tepper, 2000	ANX, DEP, STRESS	362 (NK/NK)	43	35	USA
Kivimäki *et al*., 2003	DEP	5,432 (NK/NK)	89	43	Finland
Hogh *et al*., 2005	STRESS	5,652 (356/5,296)	49	41	Denmark
Eriksen *et al*., 2006	STRESS	4,076 (NK/NK)	96	45	Norway
Hoobler *et al*., 2010	STRESS	1,167 (NK/NK)	NK	NK	USA
Finne *et al*., 2011	STRESS	1,971 (NK/NK)	64	45	Norway
Hogh *et al*., 2011	STRESS	2,154 (198/1,956)	94	33	Denmark
Lahelma *et al*., 2012	STRESS	4,911 (344/4567)	80	NK	Finland
Nielsen *et al*., 2012	STRESS	1,775 (205/1,570)	55	47	Norway
Rugulies *et al*., 2012	DEP	5,101 (5021/80)	100	46	Denmark
Johannessen *et al*., 2013	STRESS	4,816 (262/4,554)	NK	NK	Norway
McTerman *et al*., 2013	DEP	2,074 (142/1,932)	44	45	Australia
Nielsen *et al*., 2013	STRESS	741 (NK/NK)	15	45	Norway
Laine *et al*., 2014	STRESS	2,430 (2319/111)	80	NK	Finland
Laschinger and Fida, 2014	STRESS	205 (NK/NK)	89	29	Canada
Reknes *et al*., 2014	ANX, DEP	1,552 (NK/NK)	NK	33	Norway
Tuckey and Neall, 2014	STRESS	221 (NK/NK)	77	36	Australia
Einarsen and Nielsen, 2015	ANX, STRESS	1,613 (202/1,411)	54	45	Norway
Figueiredo-Ferraz *et al*., 2015	DEP	372 (NK/NK)	79	38	Spain
Gullander *et al*., 2015	DEP	5,102 (594/4,508)	75	48	Denmark
Rodríguez-Muñoz *et al*., 2015	ANX	348 (NK/NK)	62	45	Spain

Studies are ordered by year of publication and cross-sectional and longitudinal study-design.

^**A**^ This column indicates in which meta-analysis the study in the corresponding row is included: depression (DEP), anxiety (ANX), post-traumatic stress disorder (PTSD), general stress-related complaints (STRESS), burnout (BO).

### Meta-analysis on the cross-sectional association between work place bullying and mental health

The average relation between workplace bullying and mental health was *r* = .36 (95% *CI* = .32–.40, *p* < 0.001, k = 48, *N* = 115.783). Heterogeneity was substantial, 98.50%, Q(48) = 3870.44, *p* < .0001). Workplace bullying was found to be positively associated with depression (*r* = .29, 95% *CI* = .23–.34, *p* < 0.001, k = 19, *N* = 68.010), anxiety (*r* = .34, 95% *CI* = .29–.40, *p* < 0.001, k = 19, *N* = 60.802) and stress-related psychological complaints (*r* = .37, 95% *CI* = .30–.44, *p* < 0.001, k = 27, *N* = 51.683). Substantial between-study heterogeneity in outcomes was observed in the analysis with mental health as an outcome (*I* ^2^ = 98.55%; Q(48) = 3870.44, *p* < .0001) and also in the three subsequent meta-analyses (see **Table 2**).

**Table 2:** Pooled effect size estimates, between-study heterogeneity and publication-bias.

	*K*	*N*	Weighted *r* (95% *CI*)	Heterogeneity		Publication bias
				*I* ^2^	*Q*	Egger’s z
*Cross-sectional studies*						
Mental health	48	115783	0.36 (0.32–0.40) **	98.55%	3870.44 **	-1.61
Depression	19	68010	0.29 (0.23–0.34) **	97.67%	730.72 **	-0.81
Anxiety	19	60802	0.34 (0.29–0.40) **	97.71%	338.26**	0.12
General anxiety	12	57573	0.28 (0.24–0.32) **	94.40%	89.32 **	
PTSD	7	3450	0.46 (0.37–0.55) **	90.61%	63.60 **	
Stress-related complaints	27	47522	0.37 (0.30–0.44) **	98.68%	1838.95 **	-1.67
General	21	45404	0.34 (0.26–0.41) **	98.79%	1505.41 **	
Burnout	6	2118	0.51 (0.39–0.62) **	90.25%	92.11 **	
*Longitudinal studies*						
Bullying-> mental health	22	54450	0.21 (0.13–0.29) **	99.27%	7270.20 **	-1.67
Depression	7	22777	0.36 (0.17–0.56) **	99.79%	1373.02 **	
Anxiety	4	3875	0.17 (0.08–0.25) **	84.52%	27.81 **	
Stress related complaints	15	31687	0.15 (0.10–0.20) **	94.53%	240.92 **	
Mental health-> bullying	11	27028	0.18 (0.10–0.27) **	97.98%	669.33 **	-0.13
Depression	4	14298	0.13 (-0.02–0.28)	98.80%	438.90 **	
Anxiety	3	3513	0.15 (0.04–0.26) *	89.78%	26.56 **	
Stress-related complaints	7	13995	0.22 (0.12–0.31) **	97.06%	229.04 **	

* Statistical significance at *p* < .01

** Statistical significance at *p* < .001.

For depression, the type of symptoms assessed were rather consistent, yet, for anxiety and stress-related psychological complaints, we could identify two different symptom clusters. That is, 7 studies within the anxiety analysis were specifically focused on symptoms of PTSD whereas the remainder of the studies focused on anxiety in more general terms. A moderation analysis showed a significant difference between the effect size for workplace bullying and general anxiety (*r* = 0.28) versus PTSD symptoms (*r* = 0.46; Q_M_(1) = 16.09, *p* < .0001 for the difference among these two estimates). With respect to stress-related psychological complaints, 6 studies specifically focused on symptoms of burnout and the remaining studies on stress-related problems in general (e.g., worry). A moderation analysis showed a significant difference between the effect size for workplace bullying and general stress-related complaints (*r* = .34) versus burnout symptoms (*r* = .51; Q_M_(1) = 4.23, *p* = .0398). Forest-plots on these meta-analyses are provided in **S1 Fig**.

Publication bias was not observed (see **Table 2**) and sensitivity analyses showed that none of the pooled effect size estimates was unduly driven by a single study (data not shown).

We subsequently tested several possible moderating variables. The relation between workplace bullying and mental health was not affected by mean age of the participants in an individual study (Q_M_(1) = 0.15, *p* = .69), gender distribution of the individual study (percentage females; Q_M_(1) = 0.004, *p* = .95), type of work (Q_M_(2) = 3.44, *p* = .18), year of publication of the individual study (Q_M_(1) = 0.64, *p* = .42), timeframe in which the bullying was assessed (*e*.*g*., in the past 6 or 12 months; Q_M_(1) = 1.11, *p* = .29) or quality rating of the study (Q_M_(1) = 0.03, *p* = .84).

### Meta-analysis on the longitudinal association between work place bullying and mental health

Twenty-six effect sizes were available from 22 samples, derived from 21 articles, for the longitudinal analyses [12,13,17,68,71–87], (see Table 1). Mean time between the two assessments was 28 months (SD = 23). Overall, baseline exposure to workplace bullying was significantly related to subsequent mental health complaints (*r* = .21, 95% *CI* = .13–.28, *p* < .0001, k = 22, *N* = 54.450). Heterogeneity was substantial (*I* ^2^ = 99.27%; Q(21) = 7270.20, *p* < .0001). Baseline workplace bullying significantly predicted depression (*r* = .36, 95% *CI* = .16–.56, *p* < .0001, k = 7, *N* = 22.777), anxiety (*r* = .17, 95% *CI* = .08–.25, *p* < .0001, k = 4, *N* = 3.875) and stress-related psychological complaints (*r* = .15, 95% *CI* = .10–.20, *p* < .0001, k = 15, *N* = 31.687). The outcomes of these meta-analyses are provided as forest-plots in **S2 Fig**.

We subsequently tested several possible moderating variables of these relations. The longitudinal relation between workplace bullying and mental health also was consistent, and was not affected by mean age of the individual study (Q_M_(1) = 2.3090, *p* = .13), gender distribution of the individual study (percentage females; Q_M_(1) = 0.52, *p* = .47), type of work (Q_M_(2) = 4.24, *p* = .12), year of publication of the individual study (Q_M_(1) = 0.0021, *p* = .96), number of months between the two assessments (Q_M_ (1) = 0.08, *p* = .77), timeframe in which the bullying was assessed (*e*.*g*., in the past 6 or 12 months; Q_M_ (1) = 0.901, *p* = .34) or quality rating of the study (Q_M_(1) = 0.57, *p* = .45).

Additionally, a reversed association between mental health problems at baseline and exposure to workplace bullying at follow-up was detected (*r* = .18, 95% *CI* = .10–.27, *p* < .0001, k = 11, *N* = 27.028). This reversed association was observed for studies reporting on anxiety (*r* = .15, 95% *CI* = .04–.25, *p* < .01, k = 3, *N* = 3.513) and stress-related psychological complaints (*r* = .22, 95% *CI* = .11–.31, *p* < .0001, k = 7, *N* = 13.995), but was not apparent for depression (*r* = .13, 95% *CI* = -.02–.28, *p* = .096, k = 4, *N* = 14.298). Moderation analyses were not conducted due to the small number of studies. Forest-plots on these associations can be found in **S3 Fig**.

For the longitudinal studies, there was no evidence for publication bias and none of the outcomes was unduly driven by a single study.

## Discussion

Here we show, by pooling the available cross-sectional and longitudinal data (70 samples and a total of 170.233 participants) that workplace bullying is positively related to depressive-, anxiety-, and PTSD symptoms and stress-related psychological complaints. The effect size estimates on these associations (Pearson’s *r*) range from .15–.51, which is consistent with a previous meta-analysis [9]. These results indicate that workplace bullying explains about 2.25 to 26 percent of the variance in outcomes. Herewith, workplace bullying appears as a predictor of depressive-, anxiety-, and PTSD symptoms and stress-related psychological complaints that is of comparable strength as way more often studied risk predictors for stress related psychopathology such as obesity [88], sleep and exercise [89], and exposure to stressful events (e.g., job loss or a divorce [90]).

The observed associations can be explained by stress models that emphasize that prolonged periods of stress are detrimental for somatic as well as mental health [91,92]. Workplace bullying can be considered a source of prolonged social defeat stress that affects emotional well-being, likely through changes in neuroendocrine, autonomic and immune functioning [93–96]. Additionally, it is possible that the effects of workplace bullying are not specifically due to actual encounters with the bully/bullies, or that these changes are only observed during working hours. Such invasive experiences are likely to be recreated over and over again in the minds of people that are being bullied. Such perseverative, intrusive thoughts have been shown to prolong the stress response beyond actual bully experiences, thereby adding to the wear and tear effects that these experiences have [95].

In explaining the observed associations it is imperative to take two other findings from our research into account. The first of these is a significant reversed relationship between mental health complaints at baseline and exposure to workplace bullying later in time (i.e., mental health complaints predicting exposure to workplace bullying). This reversed relation was of a somewhat weaker strength as the one between workplace bullying and depressive-, anxiety-, and PTSD symptoms and stress-related psychological complaints (i.e., exposure to workplace bullying predicting mental health complaints). It should be noted though that the estimates on the strength of these relationships were based on considerable less data compared to data that was available for the prediction of mental health by workplace bullying, and thus may have been less precise. Similar findings were reported by Reijntjes et al. who found that being bullied by peers in childhood is prospectively related to changes in internalizing psychological problems (*r* = 0.18; [97]), whereas internalizing problems predicted changes in being bullied to a lesser extent (*r* = 0.08). The second finding that should be taken into account when interpreting our main results is that the effect of workplace bullying was significantly larger when stress and work related outcomes were used as outcome, that is PTSD and burn-out as compared to depression and general stress-related psychological complaints. Note that the effects of workplace bullying were statistically significant on all types of variables that were chosen as outcomes here.

### Strengths and limitations

An obvious strength of the present meta-analysis is the inclusion of a large number of cross-sectional and longitudinal studies. Furthermore, our analyses yielded highly consistent results. That is, we were able to show that the observed effects are evident for the larger part of the working population (e.g., they were evident at a similar strength in white- and blue collar work populations). Heterogeneity between the studies could also not be explained by the other moderators that we included in the analyses (mean age, gender distribution of the samples, year of publication, method of assessing bullying, quality of the study), which is similar to the findings of a meta-analysis on childhood bullying and mental health [97]. Together, the large number of studies and the consistent results suggest that our findings are reliable and generalizable to the total working population. Strengths over earlier meta-analyses include that: (I) the findings herein are based on a considerable larger amount of studies, (II) we present a comprehensive picture on a range of outcomes and the differential effect that workplace bullying may have on these, (III) the data herein are based on both cross-sectional and longitudinal studies, and (IV) assess the bi-directional link between workplace bullying and mental health problems.

Our work also carries limitations. The first and most obvious limitation is that the findings we report on can be confounded by indication. For instance, it could be that persons who are bullied at work are more likely to have been maltreated or bullied as a child, either physically or emotionally [98]. It has indeed recently been observed that 40% of the children that experienced childhood maltreatment was also the victim of bullying by peers [99]. Therefore we cannot exclude the possibility that what we observed actually are additive, or maybe even interactive effects of early adverse events and workplace bullying on mental health. In more general terms, this concept is known as *stress proliferation*: ‘adverse circumstances early in life may render an individual (psychologically or physiologically) more likely to encounter stressful events (such as workplace bullying) later in life’ [100]. Victims of bullying in childhood indeed have an increased risk of developing diminished quality of social relationships in adulthood [101]. Another limitation is that we were not able to disentangle the effects of task- versus person-related bullying (e.g., excessive monitoring versus social exclusion respectively on the outcomes of interest; [10,102]). The reason for us not to address this potentially relevant distinction is simply that no studies separated the effects of these two types of bullying with regard to outcome. A third limitation of our work is that the results of it may have been subject to *reporting bias* as the included studies all relied on self-reported data. A fourth limitation is that, although we overall report consistent findings, the findings derived from meta-regression analyses may have been under-powered since the number of studies are used as data points, and this number in general is rather small. Furthermore, it should be noted that the instrument that we used to assess individual study quality, the N-OS score, is not rigorously validated and this clearly could have limited our inability to detect associations between study quality and variation in outcomes. Notwithstanding this, the Cochrane Collaboration mentions the N-OS as the best alternative among the available instruments in the assessment of the quality of individual observational studies [103]. A final limitation is that although the results show consistent associations between workplace bullying and mental health, the magnitude of the observed associations remains weak to moderate. This suggests that other factors are at play that are protective and keep people from developing mental health problems in response to workplace bullying, such as one’s personal coping skills, or environmental factors like having a supporting family at home [104].

### Future research

Venues for future research partly come forth from the above stated limitations. A relevant future venue for work on the relation between workplace bullying and mental health is to address the possibility whether the observed effects of workplace bullying are independent of earlier exposure to stressful events or whether they actually have additive or even interactive effects (*i*.*e*., stress proliferation; [93]). Perhaps this would make it easier to address the causal nature of workplace bullying on mental health complaints. Another highly relevant venue for future work and understanding would be to study the effects of interest using longitudinal study designs that employ several measurement points over an extended period of time.

## Summary and conclusions

Based on a large pool of cross-sectional and longitudinal data, we conclude that workplace bullying is a significant predictor for subsequent mental health problems, including depressive-, anxiety-, and PTSD symptoms and other stress-related psychological complaints. By showing that mental health complaints at baseline predict later exposure to workplace bullying we also provide consistent evidence for the bi-directional nature of the association of interest. In order to intervene on the potentially damaging effects of workplace bullying it may be very important to understand the potential vicious circle of workplace bullying and mental health problems [9,12,72]. All in all our findings stress that organizations should prioritize the prevention and management of bullying at work as it has detrimental effects on the mental health of employees.

## Supporting Information

S1 PRISMA Checklist PRISMA checklist. (DOC)

S1 Fig Forest plot of the cross-sectional association between workplace bullying and mental health. (PDF)

S2 Fig Forest plot of the longitudinal association between workplace bullying and mental health. (PDF)

S3 Fig Forest plot of the longitudinal association between mental health and workplace bullying. (PDF)

S1 Table Methodological characteristics of the included studies. (DOCX)

S2 Table Newcastle-Ottawa quality assessment of the included studies. (DOCX)
