# Supplementary figures and images for: Workplace Bullying and Mental Health: A Meta-Analysis on Cross-Sectional and Longitudinal Data
Source: PLoS One. 2015 Aug 25;10(8):e0135225. doi: 10.1371/journal.pone.0135225 (PMC4549296; doi:10.1371/journal.pone.0135225)

Depression

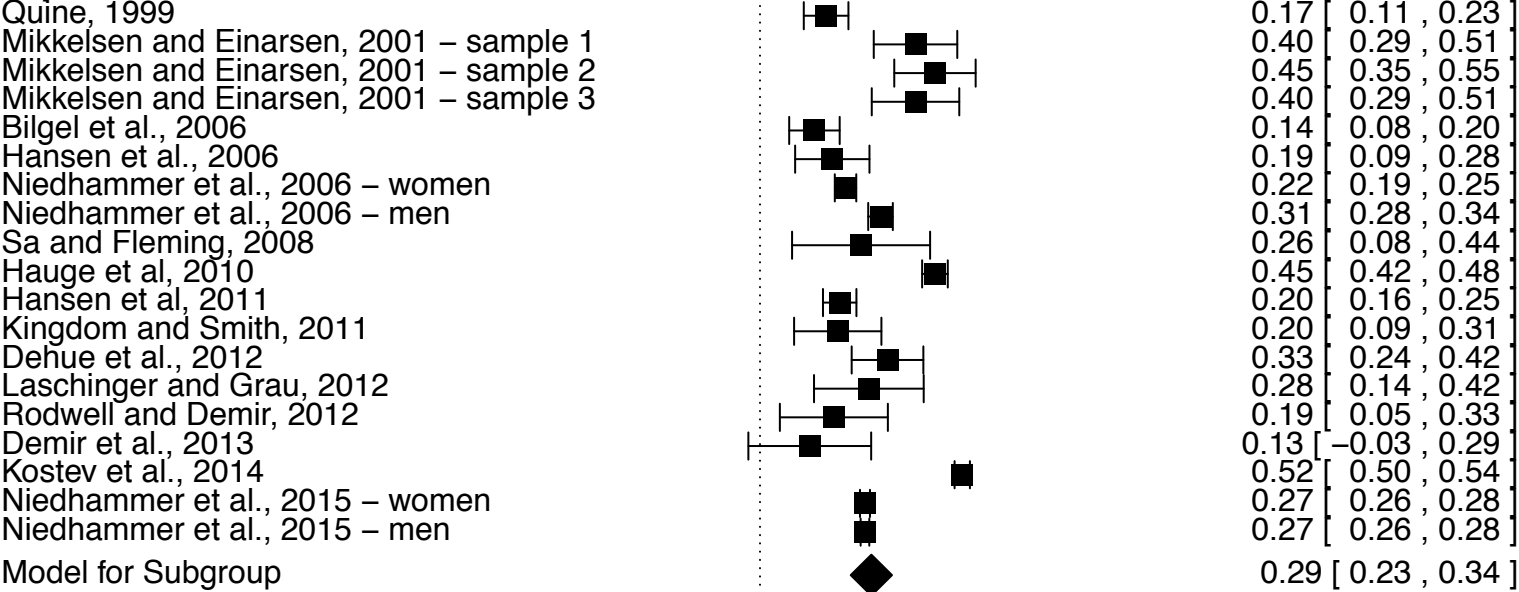

Anxiety

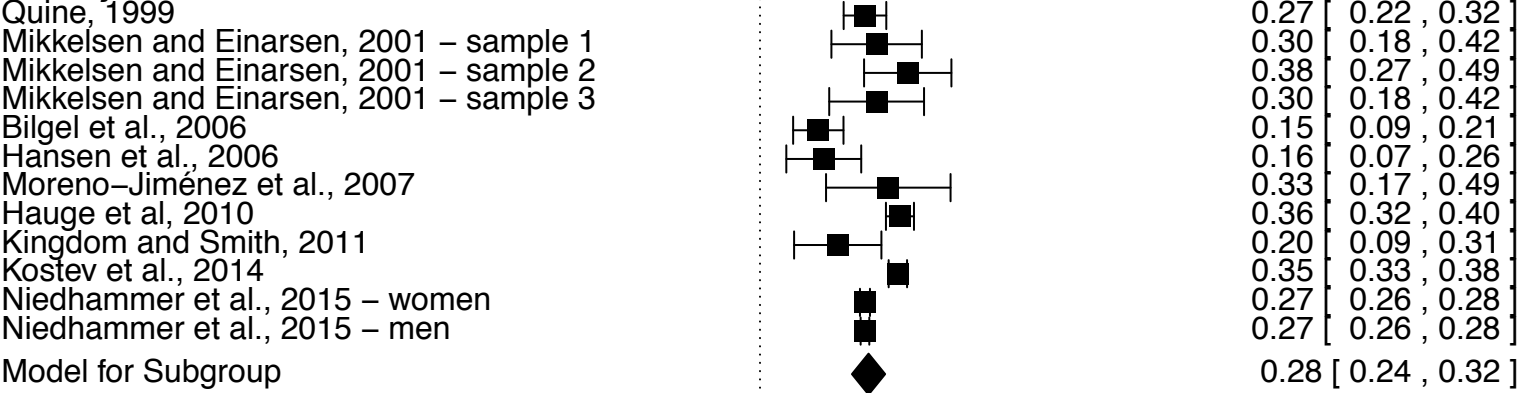

PTSD

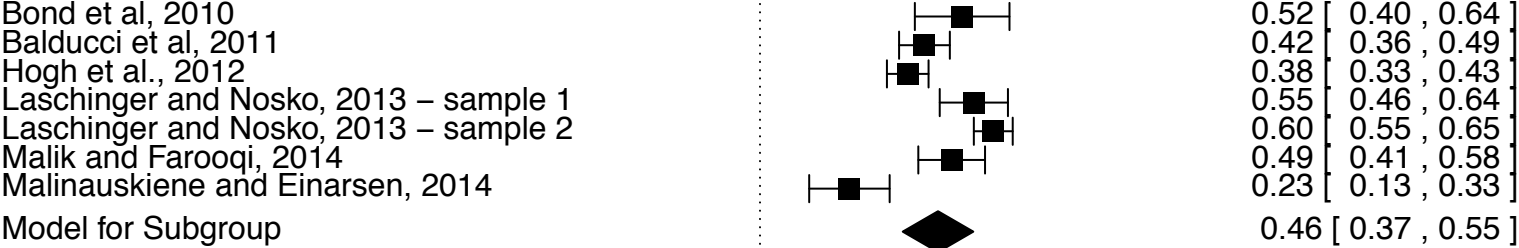

Stress-related complaints

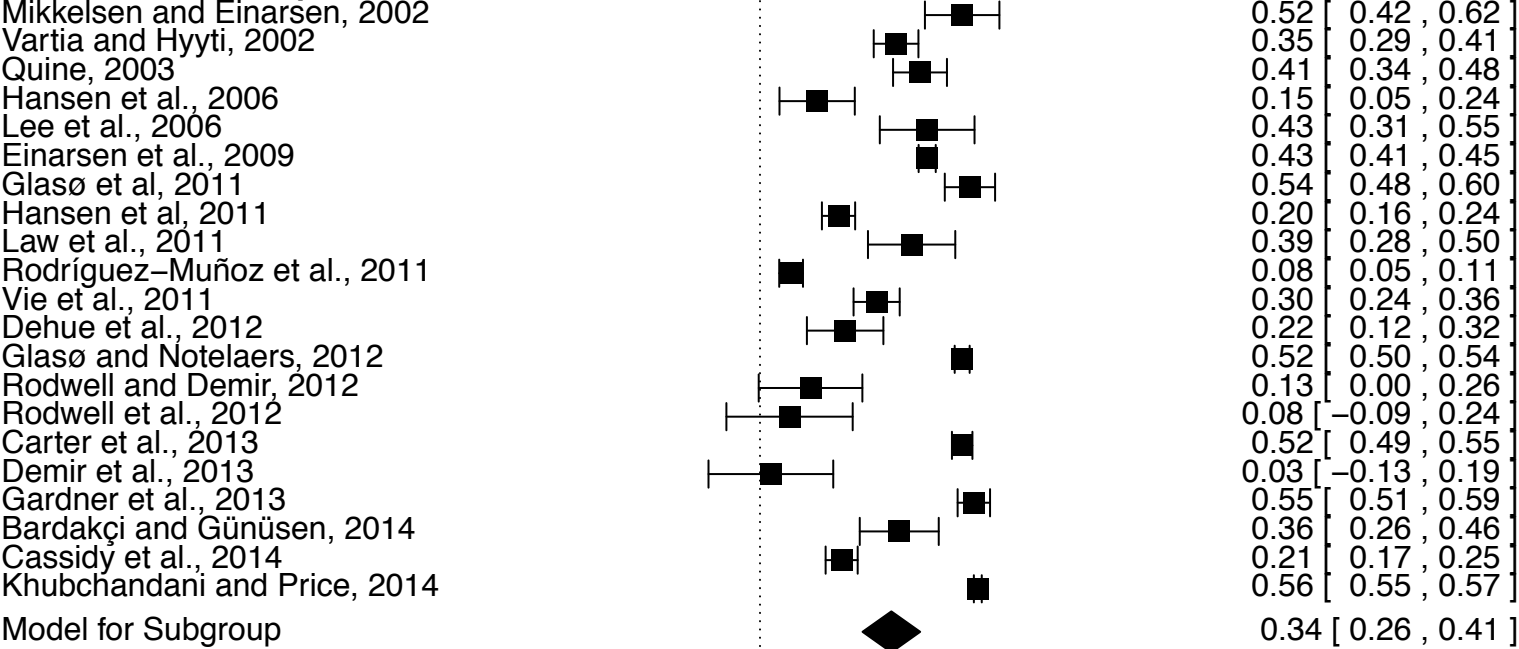

Burnout

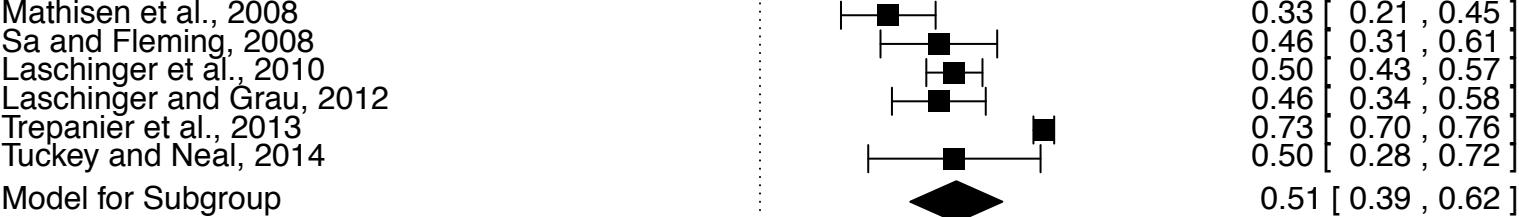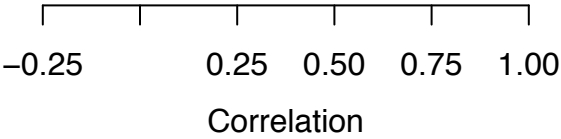

Supplement: S1 Fig — (PDF) [file pone.0135225.s002.pdf]

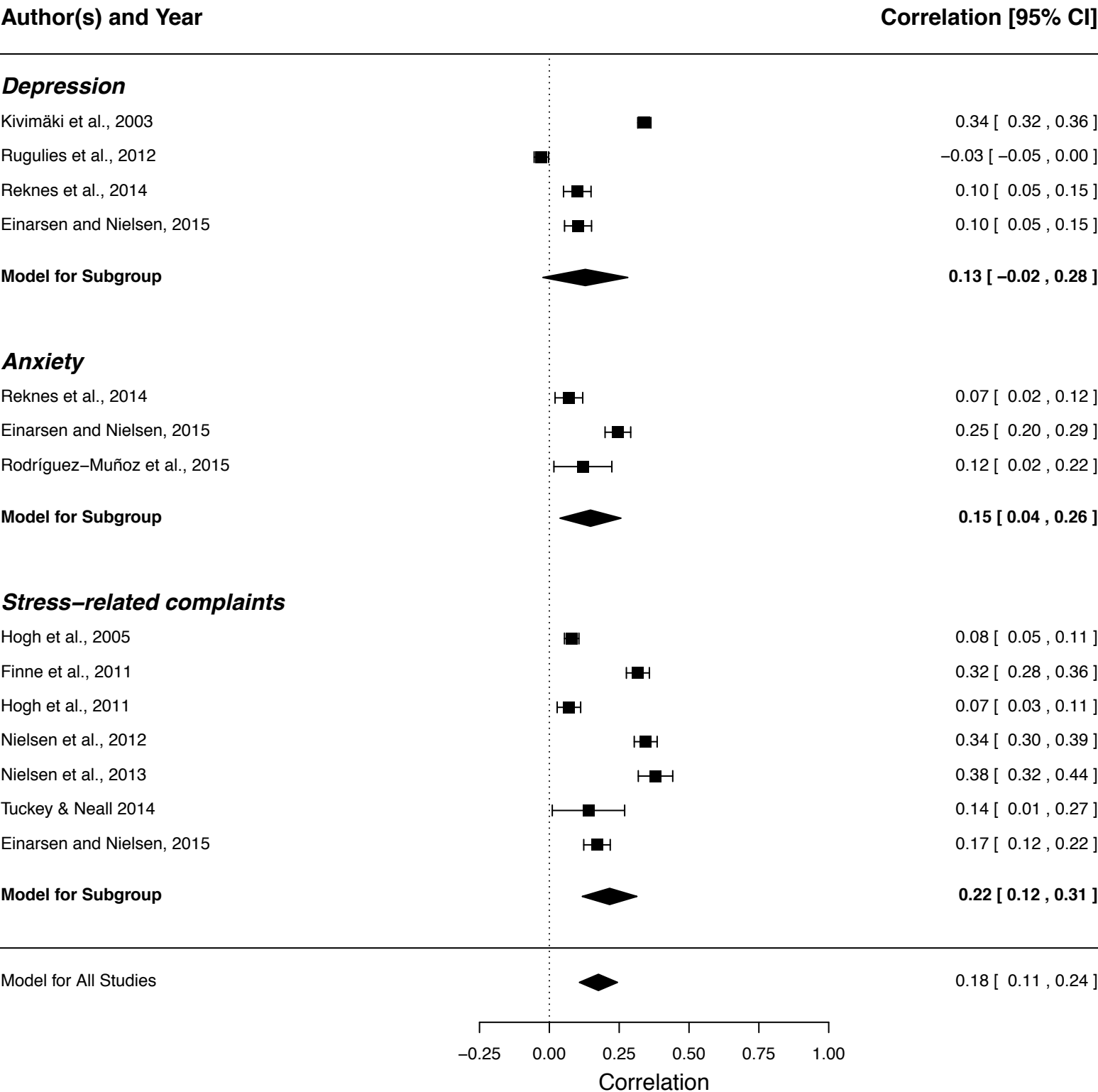

Supplement: S3 Fig — (PDF) [file pone.0135225.s004.pdf]
