# Supplementary material for: Workplace Bullying and Mental Health: A Meta-Analysis on Cross-Sectional and Longitudinal Data
Source: PLoS One. 2015 Aug 25;10(8):e0135225. doi: 10.1371/journal.pone.0135225 (PMC4549296; doi:10.1371/journal.pone.0135225)
Supplement: S2 Fig — (PDF) [file pone.0135225.s003.pdf]

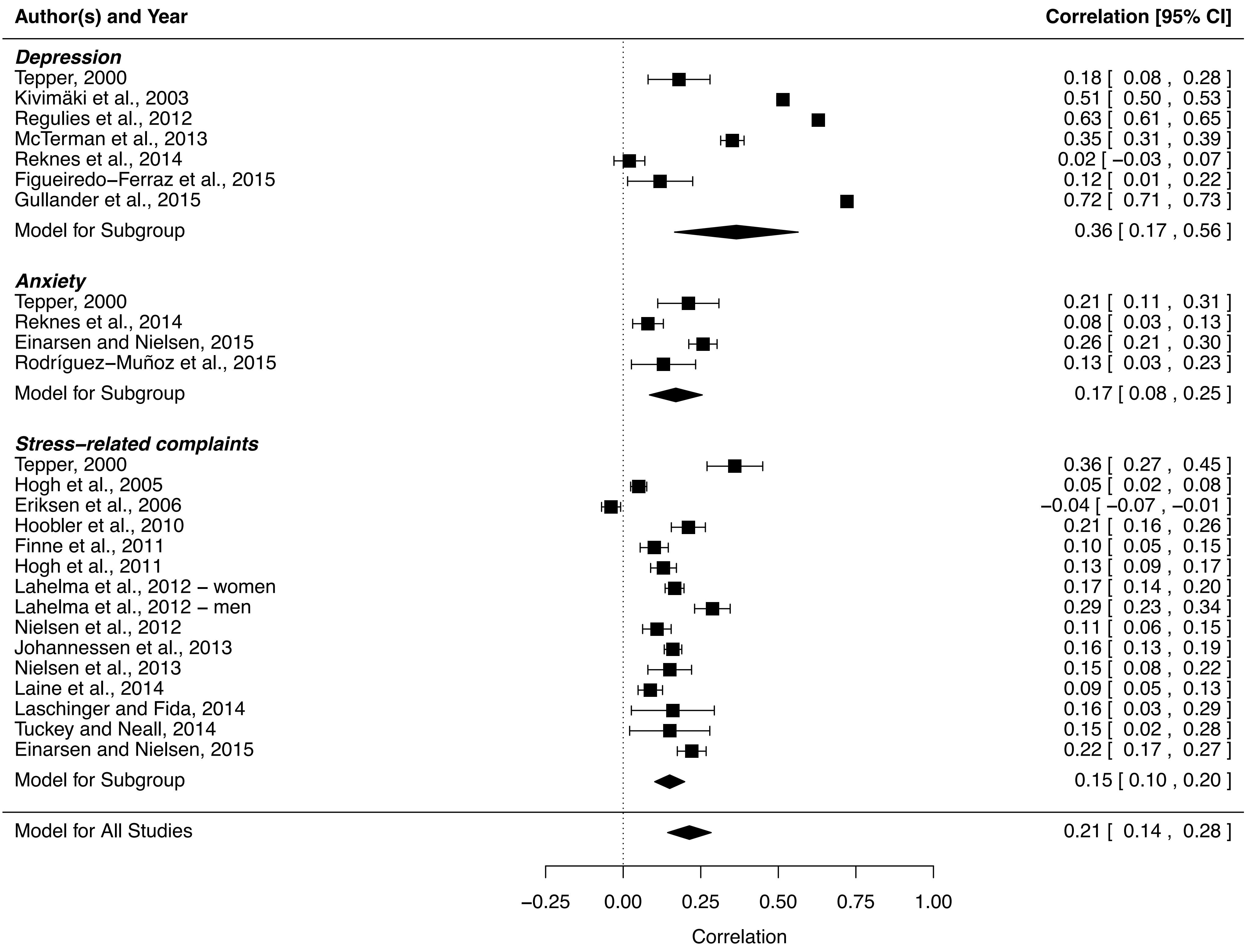
Author(s) and Year
Correlation [95% CI]
Depression
Tepper, 2000 0.18 [0.08 , 0.28]
Kivimäki et al., 2003 0.51 [0.50 , 0.53]
Regulies et al., 2012 0.63 [0.61 , 0.65]
McTerman et al., 2013 0.35 [0.31 , 0.39]
Reknes et al., 2014 0.02 [−0.03 , 0.07]
Figueiredo−Ferraz et al., 2015 0.12 [0.01 , 0.22]
Gullander et al., 2015 0.72 [0.71 , 0.73]
Model for Subgroup 0.36 [0.17 , 0.56]
Anxiety
Tepper, 2000 0.21 [0.11 , 0.31]
Reknes et al., 2014 0.08 [0.03 , 0.13]
Einarsen and Nielsen, 2015 0.26 [0.21 , 0.30]
Rodríguez−Muñoz et al., 2015 0.13 [0.03 , 0.23]
Model for Subgroup 0.17 [0.08 , 0.25]
Stress−related complaints
Tepper, 2000 0.36 [0.27 , 0.45]
Hogh et al., 2005 0.05 [0.02 , 0.08]
Eriksen et al., 2006 −0.04 [−0.07 , −0.01]
Hoobler et al., 2010 0.21 [0.16 , 0.26]
Finne et al., 2011 0.10 [0.05 , 0.15]
Hogh et al., 2011 0.13 [0.09 , 0.17]
Lahelma et al., 2012 − women 0.17 [0.14 , 0.20]
Lahelma et al., 2012 − men 0.29 [0.23 , 0.34]
Nielsen et al., 2012 0.11 [0.06 , 0.15]
Johannessen et al., 2013 0.16 [0.13 , 0.19]
Nielsen et al., 2013 0.15 [0.08 , 0.22]
Laine et al., 2014 0.09 [0.05 , 0.13]
Laschinger and Fida, 2014 0.16 [0.03 , 0.29]
Tuckey and Neall, 2014 0.15 [0.02 , 0.28]
Einarsen and Nielsen, 2015 0.22 [0.17 , 0.27]
Model for Subgroup 0.15 [0.10 , 0.20]
Model for All Studies 0.21 [0.14 , 0.28]
−0.25
0.00
0.25
0.50
0.75
1.00
Correlation
