# Supplementary material for: Workplace Bullying and Mental Health: A Meta-Analysis on Cross-Sectional and Longitudinal Data
Source: PLoS One. 2015 Aug 25;10(8):e0135225. doi: 10.1371/journal.pone.0135225 (PMC4549296; doi:10.1371/journal.pone.0135225)
Supplement: S1 Table — (DOCX) [file pone.0135225.s005.docx]

**Table S1** Methodological characteristics of the included studies

| **Author, year** | **Workplace setting ^A^** | **Blue/white collar ^B^** | **Measurement of bullying (in months) ^C^** | **Outcomes (time-span)** |
| --- | --- | --- | --- | --- |
| Quine, 1999 | Healthcare | White | Questionnaire y/n (12) | HADS |
| Mikkelsen and Einarsen*,* 2001 | General | Mix | Question scale 8 cat. (6) | HSCL-25 |
| Mikkelsen and Einarsen*,* 2002 | Manufactory | Blue | Questionnaire y/n (6) | HSCL-25 |
| Vartia and Hyyti*,* 2002 | Governmental | White | Questionnaire y/n (6) | GHQ-12 |
| Quine, 2003 | Healthcare/docters | White | Questionnaire y/n (12) | GHQ-12 |
| Bilgel *et al.,* 2006 | Governmental | White | Questionnaire y/n (12) | HADS |
| Hansen *et al.,* 2006 | General | Mix | Questionnaire y/n (6) | GHQ-12/SCL-35 |
| Lee *et al.,* 2006 | General | Mix | Questionnaire 5 cat. (6) | MBI-GS |
| Niedhammer *et al.,* 2006 | General | Mix | Question y/n (12) | CES-D |
| Moreno-Jiménez *et al.,* 2007 | General | Blue | Questionnaire 5 cat. (NK) | SADS |
| Mathisen *et al.,* 2008 | Restaurant | Mix | Questionnaire 5 cat. (6) | MBI-GS |
| Sa and Fleming, 2008 | Healthcare | White | Questionnaire 6 cat. (6) | MBI/GHQ-28 |
| Einarsen *et al.,* 2009 | General | Mix | Questionnaire 5 cat. (6) | GHQ-12 |
| Bond *et al,* 2010 | Police officers | White | Questionnaire 5 cat. (6) | PPTSD-R |
| Hauge *et al,* 2010 | General | Mix | Questionnaire 5 cat. (6) | HSCL |
| Laschinger *et al.,* 2010 | Healthcare/nurses | Blue | Questionnaire 5 cat. (6) | MBI |
| Balducci *et al,* 2011 | Administration | White | Questionnaire 5 cat. (6) | PCL-C |
| Glasø *et al,* 2011 | Transport | Mix | Questionnaire 5 cat. (6) | PANAS-NA |
| Hansen *et al,* 2011 | General | Mix | Question scale 8 cat. (6) | MDI/CPQ |
| Kingdom and Smith, 2011 | Coastgard | Mix | Questionnaire cont. (NK) | HADS |
| Law *et al.,* 2011 | General | Mix | Questionnaire y/n (6) | Kessler-10 |
| Rodríguez-Muñoz *et al.,* 2011 | General | Mix | Questionnaire 5 cat. (6) | QEEW 3 items |
| Vie *et al.,* 2011 | Transportation | Mix | Question y/n (6) | PANAS |
| Dehue *et al.,* 2012 | General | Mix | Questionnaire 4 cat. (12) | BDI |
| Glasø and Notelaers*,* 2012 | General | Mix | Questionnaire 5 cat. (6) | QEEW |
| Hogh *et al.*, 2012 | General | Mix | Questionnaire 5 cat. (6) | IES-R |
| Laschinger and Grau*,* 2012 | Healthcare/nurses | Blue | Questionnaire 5 cat. (6) | MBI/MHI-5 |
| Rodwell and Demir, 2012 | Healthcare/nurses | Blue | Question y/n (6) | CES-D/Kessler-10 |
| Rodwell *et al.,* 2012 | Healthcare | Mix | Question y/n (6) | Kessler-10 |
| Carter *et al.,* 2013 | Healthcare | Mix | Questionnaire 5 cat. (6) | GHQ-12 |
| Demir *et al.,* 2013 | Healthcare | Mix | Question y/n (6) | CES-D |
| Gardner *et al.,* 2013 | General | Mix | Questionnaire 5 cat. (6) | GHQ-12 |
| Laschinger and Nosko, 2013 | Healthcare/nurses | Blue | Questionnaire 5 cat. (6) | PTSD scan |
| Trepanier *et al.,* 2013 | Healthcare/nurses | Blue | Questionnaire 6 cat. (6) | MBI |
| Bardakçi and Günüşen*,* 2014 | Healthcare/nurses | Blue | Questionnaire 6 cat. (12) | GHQ-12 |
| **Table S1 continues on the next page** | |  |  |  |
| **Table S1 (cont.)** |  |  |  |  |
| **Author, year** | **Workplace setting ^A^** | **Blue/white collar ^B^** | **Measurement of bullying (in months)** | **Outcomes (time-span)** |
| Cassidy *et al.,* 2014 | General | Mix | Questionnaire 5 cat. (6) | GHQ-12 |
| Khubchandani and Price, 2014 | General | Mix | Question y/n (12) | Kessler-6 |
| Kostev *et al.,* 2014 | General | Mix | Y/N from physicians’ files (NK) | ICD-10 |
| Malik and Farooqi, 2014 | Healthcare/broad | Mix | Questionnaire NK (NK) | PCL-C |
| Malinauskiene and Einarsen, 2014 | Healthcare/docters | White | Question y/n (6) | IES-R |
| Tuckey and Neal, 2014 | Retail | Mix | Questionnaire 5 cat. (6) | MBI |
| Niedhammer *et al.,* 2015 | General | Mix | Questionnaire NK (NK) | HADS |
|  |  |  |  |  |
| ***Longitudinal studies*** |  |  |  |  |
| Tepper*,* 2000 | General | Mix | Questionnaire NK (NK) | CESD, MBI |
| Kivimäki *et al.,* 2003 | Healthcare/broad | Mix | Question y/n (NK) | SF-36, GHMS |
| Hogh *et al.,* 2005 | General |  | Question y/n (NK) | SF-36, MHI |
| Eriksen *et al.,* 2006 | Healthcare/nurses | Blue | Question y/n (6) | HSCL |
| Hoobler *et al.,* 2010 | General | Mix | Questionnaire 4 cat. (12) | MHI-5 |
| Finne *et al.,* 2011 | Managerial | White | Question y/n (6) | HSCL |
| Hogh *et al.,* 2011 | Healthcare/nurses | Blue | Question y/n (12) | SF-36, MHI |
| Lahelma *et al.,* 2012 | Healthcare/broad | Mix | Question y/n (6) | GHQ-12 |
| Nielsen *et al.,* 2012 | General | Mix | Questionnaire 5 cat. (6) | HSCL |
| Regulies *et al.,* 2012 | Healthcare/broad | Mix | Question y/n (12) | MDI-10 |
| Johannessen *et al.,* 2013 | General | Mix | Question y/n (NK) | 2 items |
| McTerman *et al.,* 2013 | General | Mix | Question y/n (6) | PHQ-9 |
| Nielsen *et al.,* 2013 | Offshore | Mix | Questionnaire 5 cat. (6) | HSCL |
| Laine *et al.,* 2014 | General | Mix | Question y/n (NK) | GHQ-12 |
| Laschinger and Fida, 2014 | Healthcare/nurses | Blue | Questionnaire 5 cat. (6) | MBI |
| Reknes *et al.,* 2014 | Healthcare/nurses | Blue | Questionnaire 5 cat. (6) | HADS |
| Tuckey and Neall, 2014 | Retail | Mix | Questionnaire 5 cat. (6) | MBI |
| Einarsen and Nielsen*,* 2015 | General | Mix | Questionnaire 5 cat. (6) | HSCL |
| Figueiredo-Ferraz *et al.,* 2015 | Healthcare/broad | Mix | Questionnaire 4 cat. (*NK*) | ZUNG-SR-DRS |
| Gullander *et al.,* 2015 | General | Mix | Question y/n (6) | SCAN |
| Rodríguez-Muñoz *et al.,* 2015 | General | Mix | Questionnaire 5 cat. (6) | HADS-A |
|  |  |  |  |  |

**^A^** General here indicates that the data was gathered over a broad range of workplace settings (*e.g.,* healthcare and retail)

**^B^** Mix here indicates that the data was gathered over blue- and white-collar workers (*e.g.,* craftsmen and managers)

**^C^** Indicates what kind of instrument was used to measure workplace bullying: questionnaire or single item question or an indication from physicians’ files; number of answering categories (‘yes’ versus ‘no’ or a wider range). The number between brackets represents the timeframe (in months) over which the bullying was assessed.

**Abbreviations (alphabetical):** *BDI*, Beck’s Depression Inventory; *BHC*, Bergen Health Checklist; *CES-D*, Center of Epidemiological Studies Depression scale; *GHQ-12*, General Health Questionnaire 12 item version; *HADS*, Hospital Anxiety and Depression Scale; *HADS-A*, Hospital Anxiety and Depression Scale Anxiety; *[H]SCL-25*, [Hopkins] Symptom Check list 25 item version; *ICD-10*, International Classification of Diseases version 10 World Health Organization; *IES-R*, Impact of Events Scale Revisited; *MBI-[GS]*, Maslach Burn-out inventory [General Survey]; *MDI-10*, Major Depressive Inventory, 10 item version; *MHI-5*, Mental Health 5 item version; *OSQ*, Occupational stress questionnaire; *PANAS-NA*, Positive and Negative Affect Schedule Negative Affect Scale; *PCL-C*, PTSD Checklist Civilian version; *PDS*, Post-traumatic Diagnostic Scale; *PHQ-9*, patient Health Questionnaire 9 item version; PPTSD-R, Penn Post-Traumatic Stress Disorder Revised; *PWI*, Psychological Workplace Inventory; *QEEW*, Questionnaire on the Evaluation and Experience of Work; *SADS*, Social Avoidance and Distress Scale; *SCAN* interview, Schedule for Assessment in Neuropsychiatry interview; SF-36, Short Form health survey; *WSS*, Work-ability Support Scale; *ZUNG-SR-DRS*, Zung Self Report Depression Rating Scale
