# Supplementary material for: Workplace Bullying and Mental Health: A Meta-Analysis on Cross-Sectional and Longitudinal Data
Source: PLoS One. 2015 Aug 25;10(8):e0135225. doi: 10.1371/journal.pone.0135225 (PMC4549296; doi:10.1371/journal.pone.0135225)
Supplement: S2 Table — (DOCX) [file pone.0135225.s006.docx]

**Table S2** Newcastle-Ottawa quality assessment of the included studies

|  | **Rater I** | **Rater II** | **Mean rater I and II** |
| --- | --- | --- | --- |
| ***Cross-sectional studies*** |  |  |  |
| Quine, 1999 | 5 | 4 | 4,5 |
| Mikkelsen and Einarsen*,* 2001 | 3 | 4 | 3,5 |
| Mikkelsen and Einarsen*,* 2002 | 3 | 3 | 3 |
| Vartia and Hyyti*,* 2002 | 4 | 2 | 3 |
| Quine, 2003 | 4 | 4 | 4 |
| Bilgel *et al.,* 2006 | 5 | 5 | 5 |
| Hansen *et al.,* 2006 | 3 | 4 | 3,5 |
| Lee *et al.,* 2006 | 3 | 3 | 3 |
| Niedhammer *et al.,* 2006 | 5 | 5 | 5 |
| Moreno-Jiménez *et al.,* 2007 | 3 | 2 | 2,5 |
| Mathisen *et al.,* 2008 | 6 | 4 | 5 |
| Sa and Fleming, 2008 | 3 | 3 | 3 |
| Einarsen *et al.,* 2009 | 3 | 4 | 3,5 |
| Bond *et al,* 2010 | 4 | 5 | 4,5 |
| Hauge *et al,* 2010 | 3 | 3 | 3 |
| Laschinger *et al.,* 2010 | 6 | 4 | 5 |
| Balducci *et al,* 2011 | 4 | 4 | 4 |
| Glasø *et al,* 2011 | 5 | 4 | 4,5 |
| Hansen *et al,* 2011 | 4 | 3 | 3,5 |
| Kingdom and Smith, 2011 | 3 | 3 | 3 |
| Law *et al.,* 2011 | 5 | 4 | 4,5 |
| Rodríguez-Muñoz *et al.,* 2011 | 6 | 4 | 5 |
| Vie *et al.,* 2011 | 4 | 4 | 4 |
| Brewer *et al.,* 2012 | 4 | 4 | 4 |
| Dehue *et al.,* 2012 | 4 | 5 | 4,5 |
| Glasø and Notelaers*,* 2012 | 3 | 4 | 3,5 |
| Hogh *et al.,* 2012 | 4 | 4 | 4 |
| Laschinger and Grau*,* 2012 | 4 | 4 | 4 |
| Rodwell and Demir, 2012 | 5 | 3 | 4 |
| Rodwell *et al.,* 2012 | 3 | 3 | 3 |
| Carter *et al.,* 2013 | 6 | 4 | 5 |
| Demir *et al.,* 2013 | 4 | 3 | 3,5 |
| Gardner *et al.,* 2013 | 5 | 4 | 4,5 |
| Laschinger and Nosko, 2013 | 6 | 4 | 4 |
| Trepanier *et al.,* 2013 | 4 | 4 | 4 |
| Bardakçi and Günüşen*,* 2014 | 5 | 4 | 4,5 |
| Cassidy *et al.,* 2014 | 4 | 4 | 4 |
| Khubchandani and Price, 2014 | 5 | 5 | 5 |
| Kostev *et al.,* 2014 | 7 | 6 | 6,5 |
| Malik and Farooqi, 2014 | 4 | 4 | 4 |
| Malinauskiene and Einarsen, 2014 | 4 | 4 | 4 |
| Tuckey and Neal, 2014 | 4 | 4 | 4 |
| Niedhammer *et al.,* 2015 | 5 | 6 | 5,5 |
| ***Longitudinal studies*** |  |  |  |
| Tepper*,* 2000 | 3 | 3 | 3 |
| Kivimäki *et al.,* 2003 | 6 | 6 | 6 |
| Hogh *et al.,* 2005 | 4 | 4 | 4 |
| Eriksen *et al.,* 2006 | 5 | 6 | 5,5 |
| Hoobler *et al.,* 2010 | 4 | 4 | 4 |
| Finne *et al.,* 2011 | 5 | 5 | 5 |
| Hogh *et al.,* 2011 | 5 | 4 | 4,5 |
| Lahelma *et al.,* 2012 | 6 | 6 | 6 |
| Regulies *et al.,* 2012 | 5 | 5 | 5 |
| Johannessen *et al.,* 2013 | 5 | 7 | 6 |
| McTerman *et al.,* 2013 | 5 | 6 | 5,5 |
| Nielsen *et al.,* 2012 | 3 | 6 | 4,5 |
| **Table S2 continues on the next page** |  |  |  |
|  | **Rater I** | **Rater II** | **Mean rater I and II** |
| Nielsen *et al.,* 2013 | 5 | 5 | 5 |
| Laine *et al.,* 2014 | 3 | 7 | 5 |
| Laschinger and Fida, 2014 | 5 | 5 | 5 |
| Reknes *et al.,* 2014 | 5 | 7 | 6 |
| Tuckey and Neall, 2014 | 4 | 4 | 4 |
| Einarsen and Nielsen*,* 2015 | 6 | 7 | 6,5 |
| Figueiredo-Ferraz *et al.,* 2015 | 5 | 4 | 4,5 |
| Gullander *et al.,* 2015 | 5 | 4 | 4,5 |
| Niedhammer *et al.,* 2015 | 5 | 6 | 5,5 |
| Rodríguez-Muñoz *et al.,* 2015 | 5 | 6 | 5,5 |

**Table 2.** Pooled effect-size estimates, heterogeneity and publication bias by the outcome indicated in the row.

|  | No. of comparisons | No. of participants | | Hedges’ *g* (95% *CI*) | Heterogeneity | | Publication bias |
| --- | --- | --- | --- | --- | --- | --- | --- |
|  |  | Non-bullied | Bullied |  | *I*^2^ | *Q* | Egger’s *t* |
| *Cross-sectional studies* |  |  |  |  |  |  |  |
| Depression | XXX | X,XXX | X,XXX | X.XX (X.XX – X.XX) ** | XX.X% | XX.X ** | X.X ** |
| Anxiety |  |  |  |  |  |  |  |
| PTSD |  |  |  |  |  |  |  |
| General mental health |  |  |  |  |  |  |  |
| Medication use |  |  |  |  |  |  |  |
| *Longitudinal studies* |  |  |  |  |  |  |  |
| Depression |  |  |  |  |  |  |  |
| Anxiety |  |  |  |  |  |  |  |
| PTSD |  |  |  |  |  |  |  |
| General mental health |  |  |  |  |  |  |  |
| Medication use |  |  |  |  |  |  |  |
| **^a^** Effect-size estimates were of a larger magnitude in studies that **… XXXX [e.g. took depression as an outcome measure]**  **^b^ Continuous measurements of workplace bullying [= e.g.]** yielded larger effect-size estimates as compared to **single item measurements of workplace bullying**  * Statistical significant at *P* < .05; ** Statistical significance at *P* < .01; *** Statistical significance at *P* < .001. | | | | | | | |

**Table 3.** Pearson’s and Spearman’s correlation coefficients among the outcome indicated in the columns and the potential moderating variable indicated in the corresponding row.

|  | % female | Mean age | Workplace setting | Measurement of bullying |
| --- | --- | --- | --- | --- |
| *Cross-sectional studies* | *k* = XX, *n* = X,XXX | *k* = XX, *n* = X,XXX | *k* = XX, *n* = X,XXX | *k* = XX, *n* = X,XXX |
| All | X.XX** | X.XX** | X.XX* | X.XX |
| Depression |  |  |  |  |
| Anxiety |  |  |  |  |
| PTSD |  |  |  |  |
| General mental health |  |  |  |  |
| Medication use |  |  |  |  |
| *Longitudinal studyies* | *k* = XX, *n* = X,XXX | *k* = XX, *n* = X,XXX | *k* = XX, *n* = X,XXX | *k* = XX, *n* = X,XXX |
| All |  |  |  |  |
| Depression |  |  |  |  |
| Anxiety |  |  |  |  |
| PTSD |  |  |  |  |
| General mental health |  |  |  |  |
| Medication use |  |  |  |  |
| * statistically significant at *P* < .05; ** statistically significant at *P* < .01; *** statistically significant at *P* < .001 | | | | |
